# Evaluating the maturation of mental health systems in developing countries

**DOI:** 10.1002/pcn5.109

**Published:** 2023-06-06

**Authors:** Akihiro Nishio, Toshiyuki Marutani

**Affiliations:** ^1^ Health Administration Center Gifu University Gifu Japan; ^2^ Department of Global Health, Graduate School of Health Sciences University of the Ryukyus Nishihara Cho Japan; ^3^ Health Support Center Ochanomizu University Tokyo Japan

**Keywords:** Cambodia, developing countries, duration of untreated mental illness, duration of untreated psychosis, local, mental health

## Abstract

**Background:**

Given the need for a simple tool to evaluate mental healthcare provision at the local level, we compared the duration of untreated mental illness (DUM) and duration of untreated psychosis (DUP) between rural and urban areas in Cambodia, and Cambodia's DUP was also compared with that of other countries.

**Methods:**

DUM and background data were obtained at the first consultation from 940 participants in Phnom Penh (PP), the capital city, and Siem Reap province (SR) in 2016–2017. DUP data were obtained from DUM by excluding individuals with nonpsychotic mental illnesses (e.g., mood disorders, neurotic disorders, substance use, epilepsy). Student's *t*‐test was used to compare DUM and DUP, and analysis of variance was conducted to identify associations.

**Results:**

Mean DUM significantly differed between PP (0.6 [SD 2.3] years) and SR (4.2 [5.9] years). Mean DUP was also significantly different (0.5 [2.2] years in PP vs. 4.3 [6.7] years in SR). DUM was strongly associated with DUP. The prevalence of the various disorders differed between rural and urban areas. DUM also varied by diagnosis, indicating that DUP is a better index than DUM. However, in some cases DUM can be used, given its strong association with DUP. DUP in PP was almost the same as in developed countries and was about four times longer in SR.

**Conclusion:**

DUP in rural areas is much longer than in urban areas in developing countries. Although DUP is an effective index, more data from other places and before/after interventions are required to verify it further.

## Graphical abstract

Histogram of duration of untreated psychosis (DUP) in Phnom Penh (PP) and Siem Reap (SR).

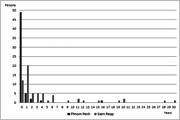

## INTRODUCTION

Health systems have not yet adequately responded to the burden of mental health disorders and, consequently, there is a large gap between the demand and provision of mental health treatment worldwide. In low‐ and middle‐income countries, between 76% and 85% of individuals with severe mental health disorders cannot receive adequate treatment.^1^ One reason for this gap might be the difficulty in implementing an effective mental health system with international collaboration, which is caused by the lack of clear evaluation criteria in mental health, such as infection rate or infant mortality in the physical health domain. The World Health Organization (WHO) has therefore launched practical tools to evaluate mental health systems, such as the WHO Assessment Instrument Mental Health Systems^1^ in 2005, Monitoring and Evaluation of Mental Health Policies and Plans in 2008,^2^ the Mental Health Gap Intervention Guide in 2010,^3^ and the Mental Health Action Plan in 2013.^4^ However, these tools are considered indicators to evaluate governmental infrastructure, such as the number of psychiatrists or the facilities situation, not the quality of mental healthcare provided at the provincial or district level. We consider there are three levels of evaluation: country level, regional level and individual level. This article aims to make the criteria to evaluate the second level. A simple and useful method for evaluating the quality of mental healthcare provision at the local level is required for that purpose.

To develop such a method, we defined good local mental healthcare by asking the question, “When someone develops a mental illness, can he or she can attend a psychiatric department in the early stage?” To elucidate the factors affecting medical accessibility for individuals with mental illness, we propose using the concept of duration of untreated mental illness (DUM), which is a measurement of the length of time between mental illness onset and the first visit to a psychiatric department. To introduce DUM, we referred to the concept of duration of untreated psychosis (DUP), which is used as an index for evaluating the prognosis of patients with psychosis.^5^ Many reports have suggested that a long DUP is a risk factor for poor long‐term mental health outcomes, and that shortening DUP might improve long‐term recovery.^6^, ^7^ Although DUP is generally used worldwide for predicting the prognosis of patients with psychosis, here we used DUM in its original meaning, as stated above.

We previously reported that DUP and DUM were not influenced by the gender, age, hospital access, education level, occupation, or economic status of individuals in a Cambodian sample,^8^ therefore it was presumed that DUP and DUM would not be related to individual but regional characteristics. DUP or DUM might therefore be used as indices to evaluate mental healthcare provision in targeted areas if we could show differences in DUP or DUM between an area that is considered to have a well‐matured mental health system and one that is not. Factors such as individuals’ knowledge about mental health, medical insurance, and clinical level in the targeted area might influence the DUM or DUP. Thus, using DUP as an objective index could allow us to measure the burden of visiting a psychiatric department within targeted areas. In our previous study regarding DUM in Cambodia^8^ we obtained data from patients who already used psychiatric services, therefore patients who had been visiting the hospital for a long time and those who had started visiting only recently were both included. Thus, DUM as measured in our previous study might not show the area's actual status, since factors such as local hospital access, patient education, knowledge of mental illness, and discrimination of psychotic disorders were dependent on the time of each patient's first visit to the psychiatric department.

In this study, we compared the DUM/DUP of new patients in an urban area (Phnom Penh, PP) and a rural area (Siem Reap provine, SR), and analyzed the relationship between DUM/DUP and patient background. Although SR is sometime classified as a urban area in Cambodia, we took data only in rural district hospitals. Thus we treated SR as representative of a rural area in this study. Furthermore, we compared the DUP identified in our study with that in other countries. Although it is very difficult to define the meaning of “urban” and “rural,” SR was considered a rural area because it is not the capital area and it has a high rate of households with an agricultural holding (>50%).^9^

## MATERIALS AND METHODS

### Setting

We conducted our survey in PP and SR, both in Cambodia, from 2016 to 2017. Cambodia is located in the southern part of the Indochina Peninsula in South‐East Asia and has a population of over 16 million. Cambodia has faced many political and economic difficulties, but following more than two decades of strong economic growth, it attained lower middle‐income status in 2015. The gross national income per capita (current international USD) was 1380 in 2018.^10^

With around 2.28 million habitants,^11^ PP is the capital of Cambodia. At the time of the study, there were four facilities that provided psychiatric consultation in PP: Khmer Soviet Friendship Hospital, Kossamak Hospital, Phnom Penh Municipal Hospital, and transcultural psychosocial organization (TPO) Clinics. Only the Khmer Soviet Friendship Hospital had in‐patient wards. However, the number of beds depended on the budget and was unstable. The fee for mental health services for outpatients was not covered by national health insurance and ranged from 5 to 40 USD in PP.

With a population of about 1.01 million in 2019,^11^ SR is the fourth largest province in Cambodia. At the time of our study, SR had one central hospital in Siem Reap City and three district hospitals in the Angkor Chum, Kralanh, and Sout Nikom districts. Only the central hospital had two psychiatrists on staff. The other three hospitals are about 2 or 3 h from Siem Reap City by car and provided psychiatric services twice a month, with doctors traveling from the central hospital in SR or other provincial hospitals. No in‐patient hospitalization settings were available for patients with psychosis in SR. Treatment was free for low‐income patients, who were recognized through the Identification of Poor Households Program.^12^ If patients were found to be extremely poor, the traffic fee (1 USD) was also covered for patients in SR. If patients were found to have middle or high economic status, they had to pay 5 USD for each visit, which included medications. The percentage of high‐income patients was only one‐third. Additionally, after our survey, this program went bankrupt. Following some changes to the system, only patients who were found to be extremely poor are presently exempt from paying treatment costs.

### Eligibility criteria and data collection

The participants included in this study were Cambodian, diagnosed with a mental, behavioral, or neurodevelopmental disorder according to the ICD‐10, and first visited a psychiatric department in one of the following hospitals: TPO Clinics in PP, or the Angkor Chum and Kralanh district hospitals in SR. Individuals who were under 20 years old and/or non‐Cambodian were excluded. DUM and other background data were collected by Cambodian nurses at the first consultation. Since DUM and DUP data were mistakenly collected using “year” as the measurement unit in PP, DUM and DUP collected using “month” as the unit in SR were converted to “year.” To conform to PP's data collection method, <1 year of DUM or DUP was considered 0 years.

### DUM and DUP

In this study, we obtained DUP from DUM by excluding the data of individuals who were diagnosed with mental illnesses without psychotic features, including mood disorders, neurotic disorders, substance use, epilepsy, and others. We compared gender, age, diagnosis, DUM, and DUP between PP and SR. DUM and DUP data were analyzed using “year” as the measurement unit, therefore if patients visited a psychiatric department 6 months after mental illness onset, DUM and DUP were counted as 0 years. We divided diagnoses into six categories: psychotic disorders, mood disorders, neurotic disorders, substance use, epilepsy, and others. Psychotic disorders included schizophrenia and other schizophrenia‐like disorders. Epilepsy is treated within psychiatric departments in Cambodia. Diagnoses were provided by a Cambodian psychiatrist, who used the ICD‐10 as the diagnostic framework.

### Ethical considerations and data analysis

The research design was approved by the Ethical Review Committee of the Graduate School of Medicine, Gifu University, in 2015 (approval No. 27‐134) and the Ethical Committee of Ministry of Health in Cambodia in 2016 (approval No. 031 NECHR). Statistical analyses were performed using JMP® version 10.0.2 (SAS Institute, Tokyo, Japan). Student's *t*‐tests were used to compare DUM and DUP between PP and SR. Analysis of variance was conducted to identify associations between DUM and DUP.

## RESULTS

Table 1 shows the comparison of patients’ DUM, DUP, and background characteristics between PP and SR. Of 367 participants in PP, 151 (41.1%) were men; of 573 participants in SR, 216 (37.7%) were men. The gender difference between PP and SM was not significant by chi‐square test. Mean (SD) participant ages were 41.7 (15.3) and 44.7 (14.3) years old in PP and SR, respectively, with a range from 20 to 75 years. This is not significantly different by Student *t*‐test. Percentages for psychotic disorders, mood disorders, neurotic disorders, substance use, epilepsy, and other disorders among participants were 15.8%, 49.1%, 14.2%, 1.6%, 4.1%, and 15.3%, respectively, in PP, and 12.7%, 26.8%, 37.7%, 6.4%, 1.8%, and 14.6%, respectively, in SR.

**Table 1:** Comparison of patients' background characteristics and duration of untreated mental illness (DUM) and duration of untreated psychosis (DUP) between Phnom Penh and Siem Reap.

	Phnom Penh	Siem Reap
Gender		
Men	151 (41.1%)	216 (37.7%)
Women	216 (58.9%)	357 (62.3%)
Mean age (SD)	41.7 (15.3) years	44.7 (14.3) years
Diagnosis		
Psychotic disorderes	58 (15.8%)	62 (12.7%)
Mood disorders	180 (49.1%)	131 (26.8%)
Neurotic disorders	52 (14.2%)	184 (37.7%)
Substance use	6 (1.6%)	31 (6.4%)
Epilepsy	15 (4.1%)	9 (1.8%)
Others	56 (15.3%)	71 (14.6%)
Mean of DUM (SD)	0.6 (2.3) years	4.2 (5.9) years**
Mean of DUP (SD)	0.5 (2.2) years	4.3 (6.7) years**
Median of DUM	0 years	2 years
Median of DUP	0 years	1 year

*Note*: The mean DUP/DUM was significantly longer in Siem Reap than in Phnom Penh (*p* < 0.0001).

Abbreviation: SD, standard deviation.

** *P* < 0.01.

Participants’ mean DUM (SD) in PP was 0.6 (2.3) years, ranging from 0 to 29, and 4.2 (5.9) years, ranging from 0 to 43, in SR. There was a significant difference in DUM between PP and SR (*P* < 0.0001). The mean DUP of participants in PP was 0.5 (2.2) years, ranging from 0 to 16, and 4.3 (6.7) years, ranging from 0 to 30, in SR. There was also a significant difference in DUP between PP and SR (*P* < 0.0001). According to the analysis of variance, DUM had a strong association with DUP (*P* = 0.0182). Mood disorders were much more common in PP, while neurotic disorders were much more common in SR. All substance users met the criteria for alcohol abuse or dependence.

Table 2 shows comparisons of DUM based on different diagnoses. The DUM for each diagnosis was six to nine times longer in SR than in PP and that tendency was also observed for psychotic disease, mood disorders, and neurotic disorders in PP and SR. However, the DUM for epilepsy and substance use did not show the same tendency. The DUM of epilepsy was long in PP and short in SR, compared with other disorders. The DUM of substance use was very short in PP and very long in SR.

**Table 2:** Relationship between diagnosis and time until first visit to a psychiatric department.

	DUM in PP (years)	DUM in SR (years)
Diagnosis		
Psychotic disease (SD)	0.5 (2.2)	4.3 (6.6)
Mood disorders (SD)	0.6 (2.4)	4.0 (6.1)
Neurotic disorders (SD)	0.7 (1.8)	4.1 (5.5)
Substance use (SD)	0.0 (0.0)	7.2 (7.3)
Epilepsy (SD)	1.0 (1.8)	1.6 (2.8)
Others (SD)	0.8 (2.8)	4.0 (4.9)

*Note*: The duration of untreated mental illness (DUM) for each diagnosis was six to nine times longer in Siem Reap (SR) than in Phnom Penh (PP). That tendency was also observed for psychotic disease, mood disorders, and neurotic disorders in PP and SR. However, the DUM for epilepsy and substance use did not show the same tendency. The DUM of epilepsy was long in PP and short in SR, compared with other disorders. The DUM of substance use was very short in PP and very long in SR.

## DISCUSSION

To our knowledge, this study is among the first to compare DUM and DUP between rural and urban areas in a developing country. In our survey, we found that the percentages for each mental illness were different between PP, which is urban, and SR, which is rural. Unexpectedly, the percentage of neurotic disorders was higher in rural areas than in urban areas. SR is an area where the violence during the Cambodian civil wars was particularly intense, therefore the effects of the war might still influence the rates of neurotic disorders, which included post‐traumatic stress disorder. We also found that DUM varied according to diagnosis. Although DUM does not require a confirmed diagnosis and is easy to obtain from a large number of samples, we consider it better to use DUP rather than DUM as an index, as DUP is not influenced by the differences between mental illnesses. However, if the targeted population is small, or the given diagnosis is not trustworthy, DUM should be used instead of DUP, as DUM has a strong association with DUP.

**Figure 1:**
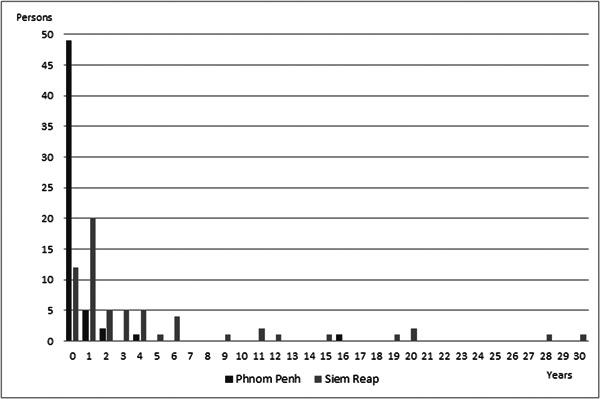
Histogram of duration of untreated psychosis (DUP) in Phnom Penh and Siem Reap. Some extremely long DUP durations were observed.

**Figure 2:**
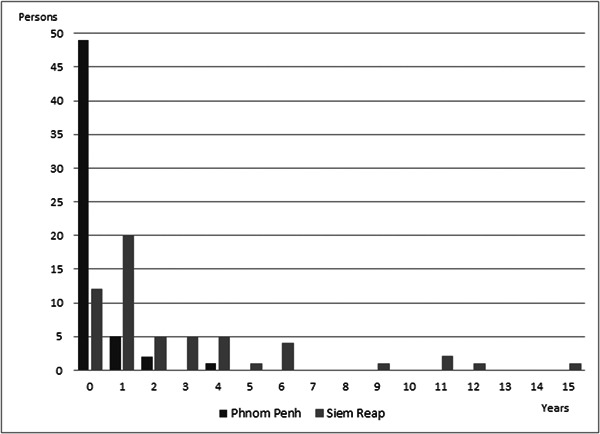
Modified version of histogram of duration of untreated psychosis (DUP) in Phnom Penh and Siem Reap. After removing 5% of extremely long DUP, the graph looks more reasonable distribution.

Although mean DUP might generally be considered to show the mental health level of a community, extremely long DUP cases were considered to be influenced not by the community mental health level but by individual characteristics. The reason for this is that some individuals might have strong beliefs or be influenced by a relative, which could prevent them from seeking mental health services for a very long period of time, therefore we should determine outlier criteria. Figure 1 shows the DUP histogram in PP and SR, which does not have a normal distribution, therefore we could not use a Smirnov–Grubbs test to determine outliers and we simply excluded the top 5% of participants as the outliers. As a result, the longest DUP was 4 years in PP and 15 years in SR. Figure 2 shows the modified version of the histogram. The modified mean DUP (SD) was 0.2 (0.7) years and 2.7 (3.3) years in PP and SR, respectively. We consider these numbers to be very reasonable for comparison. When conducting this kind of survey, it is recommended to provide a modified version of the DUP and compare it with the rough DUP to avoid serious influence from outliers.

Since the DUM and DUP data were mistakenly measured by year, we could not directly compare our DUP with that of other countries, therefore we rounded DUP in our survey to 0.5 years. We obtained the revised DUP by adding 0.5 years to our DUP and converted the measurement unit to months. Thus, we could compare this revised DUP from our survey with that of other countries. Revised mean DUP values were 12.0 (24.8) months and 57.6 (80.4) months in PP and SR, respectively.

Bora et al.^13^ conducted a meta‐analysis of DUP and selected 27 surveys from high‐income countries and Shanghai, China. According to their report, the mean DUP ranged from 9.3 to 147.2 weeks. In their survey, the quartiles of 25% and 75% were 36.9 and 77.9 weeks, respectively, therefore we can estimate the standard DUP in developed countries to be approximately 50 weeks. The DUP in PP in our survey was almost the same as that in developed countries, while the DUP in SR was about four times longer than that in developed countries. There are very few studies reporting the mean DUP in developing countries. We referred to the definition of developing countries as provided by the United Nations.^14^ Based on our research, we identified only 11 articles that showed the DUP for each country.^15^, ^16^, ^17^, ^18^, ^19^, ^20^, ^21^, ^22^, ^23^, ^24^ Table 3 shows the characteristics of the surveys and the reported DUP. Since some of the reports showed DUP using weeks as a measurement unit, we converted this to months to make the comparison easier.

**Table 3:** Characteristics of existing research and samples examining mean duration of untreated psychosis (DUP) in developing countries.

Reference	Location	Urban/rural	Subjects	Mean DUP (months)
Apiquian et al.^15^	Mexico City, Mexico	Urban	Psychosis	14.9
Oosthuizen et al.^16^	Cape Town, South Africa	Urban	Psychosis	8.2 ± 12.9
Ayres et al.^17^	São Paulo, Brazil	Urban	Psychosis	12.8
Naqvi et al.^18^	Karachi, Pakistan	Urban	Schizophrenia	14.0 ± 29.4
Sharifi et al.^19^	Teheran, Iran	Urban	Psychosis	13.0 ± 28.7
Burns et al.^20^	KwaZulu‐Natal, South Africa	Urban	Psychosis	8.8 ± 15.5
Fayes et al.^21^	Riyadh, Saudi Arabia	Urban	Psychosis	5.7 ± 4.2
Okasha et al.^22^	Egypt, Cairo	Urban	Psychosis	36.9 ± 45.3
Thirthalli et al.^23^	India, Bangalore	Urban and rural	Psychosis	22.6
Nishio et al.^8^	Siem Reap, Cambodia	Rural	Psychosis	47.0 ± 48.0
Kaminga et al.^24^	Nothan Malawi, Malawi	Rural	psychosis	71.2 ± 92.3
Nishio (this study)	Phnom Penh, Cambodia	Urban	Psychosis	12.0 ± 24.8
Nishio (this study)	Siem Reap, Cambodia	Rural	Psychosis	57.6 ± 80.4

*Note*: The DUP in rural areas was much longer than in urban areas.

As can be seen in Table 3, DUP generally ranged from 5.7 to 14 months in urban areas. Only one exception was reported: Cairo, Egypt.^22^ The mean DUP of PP in our report was 12 months, which was at the same level as other reports from urban areas in developing countries. However, there were very few reports of DUP in rural areas in developing countries. With the exception of our report, we identified only one other study, from Malawi. Since a report from India created subgroups by length of DUP, we could not obtain the raw DUP in urban or rural areas in India, but only the DUP of the whole area. Although there were few studies, we can say that the DUP in rural areas was much longer than in urban areas. In particular, the DUP in Malawi was extremely long compared with all other reports. In our study, DUP in rural areas was approximately five times longer than in urban areas. We suppose SR is privileged compared to other rural areas in Cambodia, since it has the famous Angkor Wat ruins, which attract the support of international governmental and nongovernmental organizations. Mental health services have been provided there for a long time, in contrast to the unstable supply in SR. Generally, the mental health services provided in rural areas in developing countries are extremely limited, therefore if we assessed the DUP in other rural areas, it would most likely be longer than that in SR, and might be similar to the DUP in Malawi. However, we considered our report to include a very important sample that showed the status of psychiatric services in rural areas of a developing country. Furthermore, in the future, data from other rural areas in Cambodia and other developing countries should be analyzed.

We considered DUP and DUM as possible indices to evaluate mental health systems at the local level. Basic understanding of mental health/disease, insurance systems, and the clinical reliability of hospitals/clinics in the area might influence DUM or DUP. Compared to the DUP of other countries, the DUP in our study was reasonable and might reflect the level of mental health in the targeted area. We considered that it was important to examine the supply of mental health services in the targeted area and adjust it to allow for comparison with other areas. However, we found that such supply was very unstable and we speculate that the circumstances are the same in other areas or countries. We consider that using DUP, which was simply determined from medical records or patient interviews, is not an ideal method, but a reasonable one for comparing mental health levels between areas/countries and could even be used for evaluations before/after intervention projects.

## LIMITATIONS

There were three main limitations to this study. First, the DUP data were mistakenly gathered using year as the measurement unit, instead of months, therefore the data had to be rounded up by an additional 6 months in order to be compared with the DUP of other countries. Second, we collected DUP data from a sample in a rural area. However, while SR is rural, it is unlike other rural areas in Cambodia because it contains the famous Angkor Wat ruins, therefore the data from SR might not be representative of the situation in other rural areas. However, the data was taken not in Siem Reap City but only in rural district hospitals. Thus, we considered the SR data to be representative of a rural area in this study. Third, the amount of money the patients paid was totally different in PP and SR because the national insurance system did not cover the fee for mental health services in PP. However, even if people in PP had to pay more to have psychiatric consultations in PP, they accessed the psychiatric clinics much earlier. This result matched the conclusion of the study.

Our study is among the first to compare mental healthcare levels between urban and rural areas, and with other countries using DUP as an index. In future research, it will be necessary to collect data from different places and conduct surveys before and after the implementation of activities to further verify whether DUP can be used as an index and how it should be modified.

## CONCLUSION

DUP in rural areas is much longer than in urban areas in developing countries. DUP can be a good candidate of index to mesure the mental health development.

However, more data from other places and before/after interventions is required to verify it further.

## AUTHOR CONTRIBUTIONS

Akihiro Nishio and Toshiyuki Marutani contributed to design the study. Akihiro Nishio performed the analysis of all the data and interpreted it. Akihiro Nishio wrote the manuscript. Toshiyuki Marutani supervised the study.

## ACKNOWLEDGEMENTS

We would like to express our appreciation to the staff from the SUMH Cambodia and TPO Cambodia and the psychiatrists, clinical psychologists, certified social workers, and nurses who cooperated with this survey.

## CONFLICT OF INTEREST STATEMENT

The authors declare no conflict of interest.

## ETHICS APPROVAL STATEMENT

The authors assert that all procedures contributing to this work comply with the ethical standards of the relevant national and institutional committees on human experimentation and with the Helsinki Declaration of 1975, as revised in 2008. The research design was approved by the Ethical Review Committee of the Graduate School of Medicine, Gifu University, in 2015 (approval No. 27‐134) and the Ethical Committee of Ministry of Health in Cambodia in 2016 (approval No. 031 NECHR).

## PATIENT CONSENT STATEMENT

Informed consent has been obtained from all individuals included in this study.

## CLINICAL TRIAL REGISTRATION

N/A

## Data Availability

The data supporting the findings of this study are available from the corresponding author, Akihiro Nishio, upon reasonable request.
